# Circulating biomarkers in malignant pleural mesothelioma

**DOI:** 10.37349/etat.2020.00028

**Published:** 2020-12-28

**Authors:** Giuseppe Viscardi, Davide Di Natale, Morena Fasano, Marta Brambilla, Riccardo Lobefaro, Alessandro De Toma, Giulia Galli

**Affiliations:** 1 Department of Precision Medicine, Università degli Studi della Campania “Luigi Vanvitelli”, 80131 Naples, Italy; 2 Department of Medical Oncology, Fondazione IRCCS Istituto Nazionale Tumori, 20133 Milan, Italy; 3 Department of Translational Medical Sciences, Università degli Studi della Campania “Luigi Vanvitelli”, 80131 Naples, Italy; University of Southampton, UK

**Keywords:** Mesothelioma, malignant pleural mesothelioma, liquid biopsy, biomarkers, mesothelin, miRNAs, CTC, ctDNA

## Abstract

Malignant pleural mesothelioma (MPM) is an aggressive tumor strictly connected to asbestos exposure. Prognosis is dismal as diagnosis commonly occurs in advanced stage. Radiological screenings have not proven to be effective and also pathological diagnosis may be challenging. In the era of precision oncology, validation of robust non-invasive biomarkers for screening of asbestos-exposed individuals, assessment of prognosis and prediction of response to treatments remains an important unmet clinical need. This review provides an overview on current understanding and possible applications of liquid biopsy in MPM, mostly focused on the utility as diagnostic and prognostic test.

## Introduction

Malignant pleural mesothelioma (MPM) is a tumor strictly connected with occupational and/or environmental exposure to asbestos [1]. Epithelioid subtype occurs more frequently (60%), whereas approximately 10% of MPMs are sarcomatoid and the remainder biphasic. Prognosis remains dismal as it commonly diagnosed in advanced stage and response to available medical treatments is poor. Moreover, paradigm shift of precision medicine has not yet translated into new systemic approaches [2].

Regarding mechanisms of tumorigenesis, chronic inflammation has been linked to the initiation and progression of MPM. Inhalation of asbestos fibers results in a response characterized by recruitment of macrophages and neutrophils, production of pro-inflammatory cytokines, cell proliferation and angiogenesis [3]. As result, MPM is characterized by a low mutational burden and a genomic landscape dominated by inactivation of several tumor suppressors genes, such as cyclin-dependent kinase inhibitor 2A (*CDKN2A*), BRCA1 associated protein (*BAP1*) and neurofibromatosis 2 (*NF2*) [4].

So far, radiological screenings have not proven to be effective for detecting early MPM among asbestos-exposed subjects [5]. Also differential diagnosis of MPM is sometimes very difficult. A wide tissue immunohistochemical staining, including mesothelioma-associated markers as Wilms tumor-1 (WT-1), calretinin, cytokeratin5/6 (CK5/6) and podoplanin is required for diagnosis and differentiation from pulmonary carcinoma or metastasis from other tumors [6, 7]. Since sarcomatoid histotype often does not express these markers and most commonly only stains for pan-Cytokeratin, GATA binding protein 3 (GATA-3) has been proposed as sensitive and specific biomarker for distinguishing sarcomatoid mesothelioma from sarcomatoid carcinoma of the lung [8]. Finally, BAP1 immunostaining and CDKN2A/p16 fluorescence in-situ hybridization (FISH) in effusion cytology specimens are useful in distinguishing malignant from benign mesothelial proliferation, although BAP1 and p16 are not deleted or lost in all MPM tumors [9]. In particular, *BAP1* is the most commonly mutated gene in MPM and loss of nuclear staining occurs in more than 60% of tumors [10], while homozygous deletion of *CDKN2A* is seen in 70% of epithelial MPM [11, 12].

**Figure 1.**
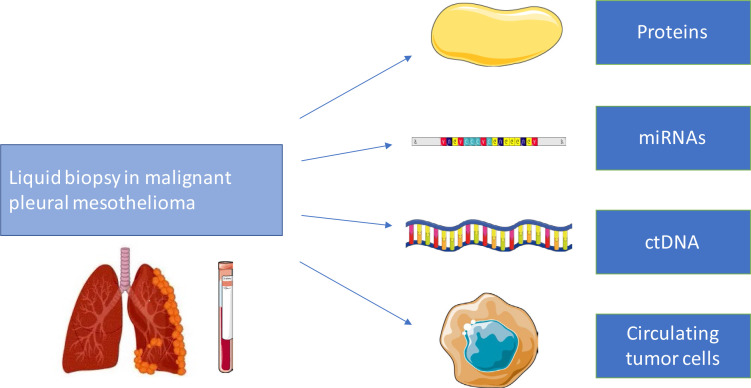
Circulating biomarkers analysis in malignant pleural mesothelioma

In this scenario, use of biological fluids, such as blood and pleural effusion, represents an ideal source of non-invasive biomarkers. Tumor cells can release into bloodstream different molecules (proteins and nucleic acids, also epigenetically modulated); circulating tumor cells (CTCs) and extracellular vesicles (EV) can be detected too (Figure 1).

So, the search for biomarkers, useful for early detection, prognosis and in the prediction of response to treatments appears as a promising field of investigation [13]. This review provides an overview on current understanding and possible developments of liquid biopsy in MPM, mostly focused on its utility as diagnostic and prognostic test.

## Proteins

A number of proteins have been evaluated in MPM over this time as diagnostic or screening markers. At the moment, mesothelin remains the most extensively studied and the only blood-based test approved by Food and Drug Administration (FDA) for detecting MPM recurrence in patients following surgery or assess response to therapies. Further candidate protein biomarkers include osteopontin (OPN), fibulin-3 (FBLN3), high-mobility group box 1 (HMGB1) protein, angiogenic factors, syndecan-1 (CD138), whereas proteomics represents a promising new approach (Table 1).

**Table 1.** Overview on proteins as biomarkers in liquid biopsy for MPM

**Author**	**Biomarker**	**Study design**	**Samples**	**Role**
Robinson et al. 2013 [16]	SMRP	MPM = 44; HS = 68 (40 AES); OPD = 160	Serum	Diagnosis: MPM *vs.* OPD (sensitivity 84%, specificity 100%)
Creaney et al. 2011 [24]	SMRP	MPM = 97	Serum	Prognosis; response to treatment
Wheatley-Price et al. 2010 [25]	SMRP	MPM = 41	Plasma	Response to treatment
Pass et al. 2005 [34]	OPN	MPM = 76; AES = 69	Serum	Diagnosis: MPM *vs.* AES (sensitivity 77.6%, specificity 85.5%)
Cristaudo et al. 2011 [39]	SMRP + OPN	MPM = 31; BRD = 204	Serum; plasma	Diagnosis: MPM *vs.* BRD (sensitivity 80%, specificity 91.2%)
Pass et al. 2012 [42]	FBLN3	MPM = 92; AES = 136; OPE = 93; HS = 43	Plasma	Diagnosis: MPM *vs.* AES (sensitivity 100%, specificity 94.1%)
		MPM = 74; OPE = 93	Pleural effusion	Diagnosis: MPM *vs.* OPE (sensitivity 83.8%, specificity 92.4%)
Creaney et al. 2014 [43]	FBLN3	MPM = 82; OPE = 71	Pleural effusion	Diagnosis: MPM *vs.* OPE (sensitivity 22%, specificity 95%); prognosis
Tabata et al. 2013 [47]	HMGB1	MPM = 61; AES = 45	Serum	Diagnosis: MPM *vs.* AES (sensitivity 34.4%, specificity 100%); prognosis
Hirayama et al. 2011 [52]	VEGF	MPM = 46; OPE = 45	Pleural effusion	Diagnosis: MPM *vs.* OPE (sensitivity 71.7%, specificity 76%); prognosis
Mundt et al. 2014 [59]	CD138	MPM = 89; OPE = 167	Pleural effusion	Diagnosis: MPM *vs.* OPE (sensitivity 74.9%, specificity 61.3%); prognosis

SMRP: soluble mesothelin related peptide; VEGF: vascular endothelial growth factor; AES: asbestos exposed subject; BRD: benign respiratory diseases; OPD: other pleural diseases; OPE: other pleural effusions

### Mesothelin

Mesothelin is a cell surface glycoprotein expressed in several tumors type, including MPM (limited to epithelioid subtype), ovarian and pancreatic adenocarcinoma, whereas normal tissues, unless mesothelial cells, exhibit very low or no expression. In particular, the term SMRP designs three isoforms, known as variant 1, 2 and 3, that can be shed by tumor cells into bloodstream [14].

Many studies attempted to assess SMRP levels in patients with MPM compared to those with non-malignant pleural disease, lung cancer and subjects exposed to asbestos, or in other cases in patients with MPM before and after treatment.

Notably, SMRP is currently the only FDA approved MPM biomarker, being Mesomark, an enzyme-linked immunosorbent assay (ELISA)-based assay, the available kit most studied for measurement of this protein in blood and pleural effusion. The cut-offs used to define an abnormal result ranged from 0.5 nmol/L to 3.3 nmol/L. Described limit of detection is 0.16 nmol/L and intra-assay imprecision (CV) 1.1–5.3%, whereas serum bilirubin, hemoglobin, triglycerides or chemotherapeutic agents do not interfere with measurement [15].

In a key study published in 2003 by Robinson et al. [16], elevated serum levels of SMRP, correlating with tumor size too, were detected in 84% of patients with MPM (*n* = 44), 2% of subjects with other cancers of inflammatory lung or pleural conditions (*n* = 120) and none of 28 controls never exposed to asbestos.

Subsequently, other heterogeneous investigations hypothesized that serum SMRP was a possible marker for diagnosis. However, a metanalysis of 16 studies showed that, employing a cut-off of 2.00 nmol/L, sensitivity ranged from 19% to 68% and specificity from 88% to 100% [17]. Despite the good specificity, other factors such as age, chronic kidney failure or obesity may be responsible of SMRP alteration [18]. Clearly, poor sensitivity limits the accuracy as diagnostic biomarker, moreover in early-stage disease and sarcomatoid mesothelioma. A cluster of MPM characterized by high score of epithelial mesenchymal transition (EMT) and poor prognosis, also shows low expression of mesothelin as consequence of promoter methylation, as previously reported for sarcomatoid type [19].

Radiologic assessment of disease and response to treatments is another issue commonly presenting in clinical management of MPM. Response evaluation criteria in solid tumors (RECIST) criteria appear inadequate, as one-dimensional measures of a tumor with a non-spherical pattern of growth are difficult [20]. Some findings support the potential value of changes in mesothelin levels for monitoring of treatment response.

High baseline SMRP levels were independently associated with poor prognosis in three prospective studies encompassing respectively 107, 96 and 100 MPM patients [21–23]. Creaney et al. [24], longitudinally assessed mesothelin serum concentrations from 97 patients diagnosed with MPM. At baseline mesothelin levels strongly correlated with tumor burden and metabolically active tumor volume as measured by fluorodeoxyglucose-positron emission tomography (FDG-PET), rather than with TNM stage. Despite the few patients examined, mesothelin levels always declined after cytoreductive surgery. Also, in patients receiving chemotherapy, there was a significant association between radiological response and decrease of serum mesothelin, as well as between change in mesothelin levels on therapy and overall survival (OS). A significant association between response outcome and relative change in plasmatic levels in 21 patients receiving systemic therapy for MPM was confirmed by Wheatley-Price et al. [25], for mesothelin, but not for OPN. However, small sample size, heterogeneity in choice of cut-off value and therapeutic interventions make difficult to draw conclusions about prognostic role of SMRP.

Lastly, given high levels of expression in MPM and limited expression in normal tissues, mesothelin represents an interesting target for development of new therapies [26]. As multiple agents are under investigation, including monoclonal antibody, antibody-drug conjugate, adoptive T cell therapy and vaccines, noninvasive biomarkers for predicting and assessing response are critical for their translation in clinical practice. As mesothelin immunohistochemistry is not predictive of response to targeted therapies, different biomarkers are to explore.

The role of megakaryocyte potentiating factor (MPF) has been investigated for this purpose. It is known that the *mesothelin* gene encodes a precursor protein that is cleaved into membrane-bound mesothelin and soluble MPF, whose serum level reflects the expression of target antigen mesothelin on tumor cells. For detection MPF assay uses a capture antibody MPF49 and a detection antibody MPF25, while mesothelin-targeted agents do not react with MPF [27]. Analyzing serum samples collected from patients enrolled in two clinical trials with anti-mesothelin immunotoxin SS1P [28, 29], Cao et al. [30], described, in presence of elevated baseline values, reduction of MPF in patients who reached partial response (PR) during systemic therapies and its association with better progression free survival (PFS) and OS. However, due to small number of patients and retrospective nature of analysis, this exploratory evidence needs to be validated prospectively in largest studies.

### OPN

OPN is an extracellular matrix protein that interacts with several surface receptors and is involved in biological processes (as bone remodeling, immune modulation and apoptosis) and pathologies (comprising infections, autoimmune disease and cancer). Several tissues and, at increased levels, tumor cells from multiple tumor types express OPN [31–33].

In the first report by Pass et al. [34], OPN serum level was associated with duration of asbestos exposure and radiographic abnormalities, showing good ability to differentiate asymptomatic asbestos-exposed individuals and early-stage mesothelioma patients (sensitivity of 84.6% and specificity of 88.4% at a cut-off value of 62.4 ng/mL). Unlike mesothelin it has been proven to rise also in patients with sarcomatoid MPM [34]. Of note, this first study did not include patients with other pleural malignancies and nonmalignant asbestos-induced pleural diseases as controls. In another investigation led by Grigoriu et al. [22], serum OPN showed insufficient specificity as diagnostic marker, being unable to discriminate between MPM and pleural metastases or benign pleural lesions caused by asbestos exposure.

In a metanalysis encompassing six studies, OPN demonstrated only a mild diagnostic sensitivity (0.65, 95% CI: 0.60–0.70) and moderate specificity (0.81, 95% CI: 0.78–0.85), being AUC under ROC curve 0.83 [35]. In this analysis serum and plasma exhibited comparable diagnostic accuracy, although OPN levels in plasma are generally higher, because of the lower stability of serum OPN caused by thrombin cleavage during the coagulation cascade [36]. As previously reported, other pre-analytical factors (as storage conditions and number of thawing) may affect OPN stability. Moreover, the choice of the ELISA kit highly influences the result of dosage [37].

Regarding evaluation of response to treatments, it has been reported that OPN levels increase together with radiological response, unlike mesothelin and MPF. This may reflect the effects of tissue remodeling on OPN levels. However, higher baseline OPN levels remained associated with shorter survival [38].

Based on the ability of serum mesothelin to diagnose epithelioid MPM and of OPN to distinguish between patients with MPM and healthy asbestos-exposed subjects, Cristaudo et al. [39], proposed to combine SMRP and plasmatic OPN in a “combined risk index” to increase both sensitivity and specificity as diagnostic marker.

### FBLN3

FBLN3 is a member of fibulin family of secreted glycoproteins and contains calcium-binding sites. It forms part of extracellular matrix and is broadly expressed on basement membranes of epithelial and endothelial cells. FBLN3 is directly involved in tissue remodeling, as well as in cell growth and tumor angiogenesis [40]. In tumorigenesis FBLN3 may have varying tissue-dependent roles [41].

Pass et al. [42], firstly reported data from 92 MPM patients and 290 controls. They reported elevated levels of FBLN3 both in plasma and pleural effusion capable to separate, with sensitivity and specificity exceeding that of any previously investigated marker, MPM patients from healthy subjects and from those with effusion due to other causes.

In spite of these results subsequent investigations have provided conflicting results. After studying a cohort of 192 patients, Creaney et al. [43], reported a sensitivity of 22% and a specificity of 95% for plasma FBLN3, although they hypothesized a superior prognostic value of FBLN3 compared with mesothelin. Data from Kirschner et al. [44], confirmed the potential prognostic value of pleural fluid FBLN3, but questioned the diagnostic role of this marker in MPM patients.

### HMGB1

HMGB1 is a member of the high-mobility group protein super-family, physiologically expressed in the nucleus of cells. Its binding to Toll like receptors (TLRs) and receptor for advanced glycation end products (RAGE) mediates the response to infectious agents and tissue injury, resulting in the promotion of inflammation and cell proliferation [45]. A key role as modulator in MPM development has also been reported. Asbestos inhalation induces mesothelial cell necrosis with release of HMGB1 in the extra-cellular space and secretion of TNF-α from inflammatory cells. Therefore, activation of NF-kB pathway contributes to transformation of mesothelial cells [46].

Tabata et al. [47], demonstrated that patients with MPM (*n* = 61) had significantly higher serum levels of HMGB1, as measured using an ELISA kit, than healthy individuals exposed to asbestos (*n* = 45). Despite a low sensitivity (34.4%), they reported very promising specificity and positive predictive value (PPV) for MPM diagnosis (100% for both at cut-off value of 9.0 ng/mL). Moreover, in this study baseline stage and serum HMGB1 levels performed as independent prognostic factors, suggesting its utility as prognostic as well as diagnostic marker.

Results from Napolitano et al. [48], suggested a role for hyperacetylated HMGB1 as diagnostic marker. Specifically, at a threshold of 2.0 ng/mL they reported sensitivity and specificity of 100% in discriminating patients with MPM compared to individuals exposed to asbestos and healthy controls. They quantified HMGB1 isoform *in vitro*, through mass spectrometry (MS) techniques, in supernatants from MPM and mesothelial cells, and found a prevalence of hyperacetylated protein in MPM. These findings are consistent with a passive release of nonacetylated HMGB1 from nucleus of necrotic cells after asbestos-induced necrosis, and active secretion of hyperacetylated isoform from cytoplasm of transformed cells.

### Angiogenic factors

VEGF is recognized as a critical regulator of vascular permeability, endothelial cell proliferation and angiogenesis. VEGF expression is well documented in MPM too, acting as autocrine growth factor and mitogen for mesothelial cells [49, 50].

As serum VEGF levels are reported to be affected by clotting through its release from platelets, a critical issue is the choice of optimal specimen for its measurement. VEGF levels in plasma are low and close to limits of ELISA sensitivity. Since platelets reservoir of VEGF may have a role in neoangiogenesis and metastasis formation, dosing VEGF from serum may improve the clinical value as biomarker [51].

Using an ELISA kit, Hirayama et al. [52], reported that patients with MPM (*n* = 46) had significantly higher pleural effusion VEGF levels than those with non-malignant pleuritis (*n* = 25) or malignant pleural effusion from lung cancer (*n* = 20, sensitivity 71.7% and specificity 76.0%, cut-off 2, 000 pg/mL). Following 28 patients with MPM, they described a significant correlation between pleural effusion VEGF levels and survival. Furthermore, serum and pleural effusion levels among 16 MPM patients were significantly closely correlate. Likewise, Yasumitsu et al. [53], reported a significant correlation between high serum VEGF concentration and worse survival (cut-off at 460 pg/mL), and the usefulness of serum VEGF to differentiate MPM patients (*n* = 51) and those with asbestosis or pleural plaques (*n* = 29). Despite Fiorelli et al. [54], observed no significant differences between malignant effusions from lung cancer (*n* = 11) and mesothelioma (*n* = 13) in VEGF levels, their data supported the use of this marker in ruling out malignancy in diagnostic work up of pleural effusion, showing that median VEGF level was significantly higher in malignant (*n* = 49) than that in benign exudates (*n* = 15).

Of interest, VEGF-targeted therapies, including bevacizumab, have been extensively investigated in MPM, but no predictive biomarkers have been identified [55]. In the MAPS trial baseline serum VEGF levels were available in 372/448 (83%) of patients. Prognostic role was confirmed, but the interaction between treatment arms (bevacizumab containing arm *vs.* chemotherapy-alone) and VEGF levels was not significant for PFS and OS [56]. Similarly, none of potential explored biomarkers were predictive of response to nintedanib in the LUME-Meso trial [57].

### CD138

CD138 is a cell surface glycoprotein that regulates biological processes as cell proliferation, migration and angiogenesis trough interaction with growth factor receptors and integrins. Its extracellular domain is shed into body fluids after cleavage by metalloproteinase and carries attachment sites for heparan sulfate chains [58].

In a study by Mundt et al. [59], pleural fluid, but not serum levels, distinguished between benign and malignant pleural effusion, from both metastatic carcinoma and pleural mesothelioma (sensitivity of 74.9%, specificity of 61.3% at the cutoff of 65.7 ng/mL). Furthermore, low levels of shed CD138 in pleural effusions predict a more favorable prognosis in patients with pleural malignancies. However, the discriminatory power of this marker seems to be insufficient to use as a sole diagnostic marker.

### Proteomics

The investigation of panels of simultaneously measured biomarkers represents an alternative to ELISA assays, which allow the study of a single candidate biomarker at time. Proteomics approach, through comparison of protein expression profile between normal samples and disease affected ones, can provide detection of new biomarkers [60]. Majority of methods for proteomics analysis are based on MS [61].

Cerciello et al. [62], used selected reaction monitoring (SRM) assay technology to evaluate the surfaceome-derived MPM biomarkers in serum samples of MPM subjects and controls. They identified a panel of seven glycopeptides, including mesothelin, who performs better than mesothelin alone in detecting MPM *vs.* non-small cell lung cancer (NSCLC) or pleural benign disease (sensitivity 91% *vs.* 74%, specificity 78% *vs.* 74%).

Kao et al. [63] identified secreted protein acidic and rich in cysteine (SPARC) as candidate prognostic plasmatic biomarker in a discovery series of 12 MPM patients through an isobaric tags for relative and absolute quantitation (iTRAC) analysis [64]. SPARC is expressed in both tumor cells and cancer-associated fibroblast [65]. In the extracellular matrix itcan modulates the interaction between cells and microenvironment, performing a role in tumorigenesis. Its prognostic role was reported in a larger retrospective series of 97 patients, finding that median survival was higher in patients with low SPARC compared with those with high SPARC (19.0 months *vs.* 8.8 months, *P* = 0.01) [63].

The slow off-rate modified aptamers (SOMAmers) proteomic technology has been used by Ostroff et al. [66] to analyze serum from 117 MPM cases and 142 high risk control patients and screen over 1, 000 protein. So, they discovered and validated a 13-biomarker panel for detection of MPM in the asbestos-exposed population (sensitivity/specificity 94%/91%, accuracy of 92%). Interestingly, SOMAmer panel includes proteins that are involved in biological processes as inflammation, cell growth regulation and cellular adhesion [67–68].

## MicroRNAs (miRNAs)

miRNAs are non-coding RNA, 20–25 nucleotides long, that regulate gene expression at post-transcriptional level by binding the 3′-untranslated regions of their messenger RNA (mRNA) targets and inhibiting translation or inducing cleavage [69].

As expected, miRNAs are involved in main human diseases, ranging from cardiovascular disorders to cancer. In tumorigenesis miRNAs can act as either tumour suppressors or oncogenes, as several targets correspond to a single miRNA. Genome-wide profiling demonstrates that miRNA expression signatures are associated with tumor type and clinical outcomes. So, miRNAs could be potential candidates for diagnostic and prognostic biomarkers, or therapeutic targets tools [70].

miRNAs are secreted into the liquid fraction of peripheral blood or other body fluids inside exosomes or in vesicle-free forms, associated with high density lipoproteins (HDL) or Argonaute proteins [71]. Therefore, these molecules can be extracted and profiled.

During the discovery phase high throughput techniques, as microarray hybridization, enable the identification of a wide number of miRNAs. Quantitative techniques, as real-time reverse transcription quantitative polymerase chain reaction (RT-qPCR) or next generation sequencing (NGS) are used during the following validation phase [72]. The choice of an appropriate normalization method, able to reduce analytical variability, represents another important aspect in miRNA quantification for clinical implementation [73].

Regarding MPM patients, many studies investigated circulating miRNA profile with the aim of identifying markers for early detection, differential diagnosis and prognosis [74] (Table 2).

**Table 2.** Overview on miRNA as biomarkers in liquid biopsy for MPM

**Author**	**Biomarker**	**Study design**	**Samples**	**Role**
Santarelli et al. 2011 [77]	miR-126	MPM = 44; AES = 196; HS = 55	Serum	Diagnosis: MPM *vs.* AES (sensitivity 73%, specificity 74%)
Kirschner et al. 2011 [84]	miR-625	MPM = 30; AES = 10	Serum	Diagnosis: MPM *vs.* AES (sensitivity 70%, specificity 90%)
Weber et al. 2012 [88]	miR-103a	MPM = 23; AES = 17	Blood	Diagnosis: MPM *vs.* AES (sensitivity 83%, specificity 71%)
Weber et al. 2017 [81]	miR-132	MPM = 21; AES = 21	Plasma	Diagnosis: MPM *vs.* AES (sensitivity 86%, specificity 61%)
Cavalleri et al. 2017 [87]	miR-103 + miR-30e EVs	MPM = 23; AES = 19	Plasma	Diagnosis: MPM *vs.* AES (sensitivity 95.5%, specificity 80%)

In 2009 Guled et al. [75], were among the first to compare miRNA profile in MPM cells and normal human pericardium using microarrays. They identified 12 over-expressed (let-7b, miR-30b, miR-32, miR-195, miR-345, miR-483-3p, miR-584, miR-595, miR-615-3p, and miR-1228) and 9 down-regulated miRNAs (let-7e, miR-7-1, miR-9, miR-34a, miR-144, miR-203, miR-340 and miR-423, miR-582) in neoplastic cells. Among those over-expressed, miR-30b, miR-32, miR-483-3p, miR-584, and miR-885-3p regulate the tumor suppressor genes *CDKN2A* and *NF2*, whereas downregulated miRNAs such as miR-9, miR-7-1 and miR-203 have as targets the oncogenes hepatocyte growth factor (*HGF*), platelet derived growth factor subunit A (*PDGFA*), epidermal growth factor (*EGF*) and Jun oncogene (*JUN*). Of interest, the majority of these miRNAs are located in chromosomal regions already known as altered in MPM, such as 8q24, 1p36, and 14q32 [76].

As reported below, among those studied in MPM patients, more evidence emerged about the role as possible circulating biomarkers of miR-126-3p, miR-103a-3p and miR-625.

In 2011 Santarelli et al. [77] obtained a miRNA signature by analyzing biopsies collected at diagnosis from MPM patients, compared to healthy human mesothelium. Among eight significantly downregulated miRNAs, miR-126 acts as tumor suppressor by decreasing the translation of vascular endothelial growth factor-A (VEGF-A) mRNA. Evaluation of serum samples obtained from 44 MPM patients, 196 asbestos-exposed and 55 HS confirmed higher concentrations of SMRP and VEGF in MPM patients. On the other hand, low levels of MiR-126 correlated with high concentration of VEGF and discriminated high risk individuals from HS (sensitivity 60%, specificity 74%) and MPM patients (sensitivity 73%, specificity 74%) [77].

In another contribution the same group demonstrated that a “3-biomarker classifier”, based on serum levels of SMRP, miR-126 and methylated thrombomodulin promoter (Met-TM), improved the differential diagnosis of MPM from asbestos-exposed subjects and healthy controls, when compared to SMRP alone [78].

Under expression of miR-126 in serum was confirmed in the study of Tomasetti et al. [79], capable to discriminate MPM patients from those with NSCLC or HS, using relative qRT-PCR. Conversely, miR-126 levels did not differentiate NSCLC patients from controls. In this study, results were normalized to small nuclear RNA U6 as endogenous control and the cel-miR-39 as exogenous control. The low expression of miR-126 was also associated with a worse prognosis, corroborating the clinical meaning of this biomarker. However, dysregulation of U6 levels in different conditions limits its potential use for normalization of circulating miRNA analysis. For instance, U6 levels were significantly upregulated in sera of patients with chronic inflammatory diseases [80].

Weber et al. [81], showed different expression levels of circulating miR-132 between mesothelioma patients and asbestos-exposed controls. For discrimination, sensitivity of 86% and specificity of 61% were calculated. Combining miR-132 with the previously described miR-126, sensitivity of 77% and specificity of 86% were obtained.

More recently, Bononi et al. [82], found three miRNAs (miR-197-3p, miR-32-3p and miR-1281) to be upregulated in MPM patients compared to HS and workers ex-exposed to asbestos (WEA), and the latter also upregulated in WEA compared to HS. Instead, the down regulation of miR-126 in serum was not confirmed. In this investigation MiR-3665, an endogenous stable microRNA (MiR-3665), was also proposed as suitable normalizer. Forkhead box O3 (*FOXO3*), a key gene promoting apoptosis, is reported as a target of miR-197. It has also been published that miR-32-3p acts as oncogene down-regulating phosphatase and tensin homolog (*PTEN*) and targeted B-cell translocation gene 2 (*BTG2*) [83].

Kirschner et al. [84], firstly reported elevated levels of a circulating miRNA with potential value as biomarker, describing, through a microarray analysis, 15 miRNAs upregulated in MPM patients compared with controls. In fact, through qRT-PCR, they demonstrated higher levels of miR-625-3p in the serum of MPM patients (*n* = 30) compared to AES (*n* = 10, sensitivity 70%, specificity 90%). However, these data were normalized against miR-16, which is known to be altered in MPM and influenced by sample haemolysis [85]. In a study aimed to assess the effects of miR-625-3p on thyroid cancer cells it appears to promote proliferation, migration and invasion by enhancing the expression of astrocyte elevated gene-1 (*AEG-1*) and activating downstream Wnt/β-catenin and Janus kinase (JNK) pathways [86]. Finally, diagnostic approaches different from those described above were proposed in other works.

In 2017 Cavalleri et al. [87], using an OpenArray method, identified a new miRNA signature in exosomes isolated from plasma. More specifically, a combination of miR-103a-3p and miR-30e-3p discriminated MPM and WEA (sensitivity 95.8%, specificity 80%).

In two consecutive works Weber et al. [88], quantified miRNAs in cellular fraction of peripheral blood. In the first study, downregulated miR-103 showed sensitivity of 83% with specificity of 71%, and sensitivity of 78% with specificity of 76% for discrimination of MPM from AES and the general population, respectively. In the second investigation, to enhance the performance of this biomarker the authors suggested a combination of miR-103a-3p and mesothelin to differentiate MPM and WEA (sensitivity 95%, specificity 81%) [89].

## Circulating tumor DNA (ctDNA)

Circulating free DNA (cfDNA) is released from healthy and cancerous tissues undergoing apoptosis or necrosis. ctDNA originates from tumor cells and carries somatic mutations, representing only a small fraction of cfDNA.

Notably, cfDNA demonstrates a wide potential as biomarker in oncology for multiple indications including staging and prognosis, monitoring response to treatments or minimal residual disease and identification of acquired resistance mechanisms to drugs [90].

The degree of cfDNA integrity, called integrity index, is based on the ratio between long and short cfDNA fragments and has been investigated as biomarker. Sriram et al. [91], found that pleural fluid DNA integrity index was higher in MPM (*n* = 52) than in benign effusions (*n* = 23). In particular, an high pleural fluid DNA integrity index exhibited a PPV of 81% in detecting MPM in patients with cytology-negative pleural effusion, suggesting its usefulness to guide more invasive procedures in this population (Table 3).

**Table 3.** Overview on various biomarkers in liquid biopsy for MPM

**Author**	**Biomarker**	**Study design**	**Samples**	**Role**
Santarelli et al. 2015 [78]	Methylated TM	MPM = 45; AES = 99; HS = 44	Serum	Diagnosis: MPM *vs.* controls (sensitivity 60%, specificity 81%)
Sriram et al. 2014 [91]	DNA integrity index (+ citology)	MPE = 52; OPE = 23	Pleural effusion	Diagnosis: MPM *vs.* OPE (sensitivity 81%, specificity 87%)
Yoneda et al. 2019 [97]	Podoplanin	MPM = 15	Blood	Diagnosis: CTC-chip *vs.* CellSearch (sensitivity 63.3%–64.5%); prognosis

TM: thrombomodulin

New perspectives have been opened afterwards the demonstration of the presence of ctDNA in treatment naïve MPM patients. Hylebos et al. [92], performed whole exome sequencing (WES) on paired germline and tumor DNA of ten MPM patients to identify cancer-specific variants of interest. Hence, using droplet digital PCR (ddPCR), they confirmed the presence of selected mutations in serum samples of three out of five treatment naive patients (detection rate 60%) at allelic fractions ranging from 0.28 to 0.9%. On the other hand, the absence of detectable tumor specific alterations in cfDNA of patients who received chemotherapy is interesting in view of the potential use of ctDNA as a biomarker for treatment response assessment.

However, broad implementation of ctDNA analysis in daily clinical practice requires validation of these data as well as use of accessible and cost-effectiveness technique.

## CTCs

CTCs are cells shed by solid tumors and circulating in the bloodstream. As CTCs play a key role in development of distant metastases, they could provide useful information in a variety of malignancies. However, their identification requires highly sensitive techniques, as they are present in a very small numbers [93].

Among various systems developed, CellSearch is the only FDA-approved for clinical purpose. It detects cells expressing epithelial cell adhesion molecule (EpCAM) through magnetic particles coated with specific antibodies [94]. As important limitation, CTCs without EpCAM expression, such as those exhibiting EMT or of non-epithelial origin, may not be recognized [95]. Accordingly, CellSearch presents a low sensibility for identification of CTCs for MPM.

To overcome this issue a CTC-chip system coated with an anti-podoplanin antibody has been developed [96]. In peripheral blood drawn from 15 MPM patients, the CTC-chip showed better performance to detect CTCs in all pathological subtypes over CellSearch (68.8% *vs.* 6.3%; *P* < 0.001), and the capability to predict unresectable disease (area under the ROC curve, 0.851). Setting a cut-off value of 2 cells/mL, an higher CTC count was also significantly associated with worse prognosis, and count decrease was demonstrated after surgical resection or tumor shrinkage [97] (Table 3).

## Epigenetic biomarkers

Epigenetic modifications occurring during tumor evolution can be detected in fluids and serve as potential biomarkers [98]. In asbestos-induced carcinogenesis, reactive oxygen species (ROS) formation is responsible of gene promoter methylation, through poly(ADP-ribose) polymerase 1 (PARP1) and DNA (cytosine-5) methyltransferase 1 (DNMT1) recruitment [99]. Interestingly, epigenetic biomarkers could represent novel therapy targets.

Nocchi L et al. [100], combined two epigenetically regulated markers (miR-126 and TM) with SMPR. In fact, it has been reported that, in MPM tissue, *TM* gene expression can be silenced through epigenetic mechanisms and, similarly, hypermethylation of miR-126 promoter region is responsible for its downregulation [101]. Despite the poor sensitivity (60%), the authors reported that circulating methylated TM DNA significantly discriminated MPM patients from controls with high specificity (82%), thus complementing the performance of miR-126 and SMRP alone for MPM detection [78] (Table 3).

More recently, Guarrera et al. [102], using a genome-wide methylation array, found differential methylation patterns of selected CpGs in DNA extracted from white blood cells (WBC) between MPM patients (*n* = 163) and controls (*n* = 137). The most hypo-methylated single-CpG is located in a gene coding for a transcription factor, namely Forkhead-box K1 (*FOXK1*), which directly interacts with BAP1. Significantly, FOXK1 maps to chromosome 7p22.2, one of the genomic areas described as associated to MPM [103], whereas somatic and germline mutation of *BAP1* is reported in MPM [10, 104].

## Exosomes

Exosomes are small nano-EV derived from the endosomal pathway and released from normal and cancer cells. Their presence in body fluids makes them potential novel biomarkers for early diagnosis and prognostication, as well as therapeutic targets [105].

The protein composition of *in vitro* produced exosomes has been studied using MS, demonstrating that exosomes derived from MPM tumour cells contain select miRNAs or cargo proteins known to be associated with angiogenesis, cell migration, metastasis, and immunoregulation [106]. Exosomes present in pleural fluid may have various cellular origins, as highlighted through proteomic analysis of malignant effusion [107].

A current limitation to adopt them as biomarkers is represented from the lack of standardization of techniques required for isolation (based on differential centrifugation) and characterization (including flow cytometry, proteomic and MS analysis).

## Immunophenotype of circulating cells

A great advantage of multiplex assay for flow cytometry is ability of measuring a large number of analytes at the same time reducing time of analysis, costs and sample volume [108].

A comprehensive study of the immune signature of MPM may contribute to understanding the immune-evasion mechanisms related to tumor growth, concurring to differential diagnosis and prognostic stratification.

Using multiparametric flow cytometry, Salaroglio et al. [109], analyzed simultaneously immune infiltrate of either pleural fluid and biopsy specimens of pleural tissue from 275 patients with pleural effusion of unknown cause. So, they described an immune signature discriminating MPM from pleuritis and from pleural metastases, identifying higher CD8 T cells and Treg cells within pleural fluid as specific biomarkers of MPM. The immune phenotype of pleural fluid cells had no prognostic significance, whereas expression of programmed death 1 (PD-1), lymphocyte activating 3 (LAG-3), and T cell immunoglobulin and mucin domain 3 (TIM-3) in tumor-infiltrating lymphocytes (TILs), but not in T cells in pleural effusion, correlated with lower OS [109].

## Discussion

MPM is a challenging malignancy to treat as early diagnosis is difficult and effective treatments are lacking. Recently, The Cancer Genome Atlas (TCGA) Research Network conducted a comprehensive integrated genomic study of MPM. A novel subtype characterized by extensive loss of heterozygosity, expression of the immune checkpoint regulator V-domain Ig suppressor of T cell activation (VISTA) and a comprehensive analysis of *BAP1* mutations emerged as key findings [12].

Disappointingly, the deeper knowledge of MPM biology has not yet translated into the validation of non-invasive biomarkers that can assist in diagnosis (identifying patients with all subtypes of MPM and predicting its development in asbestos-exposed subjects), definition of prognosis and monitor treatments response. The search for circulating biomarkers has been ongoing from 1990s. As several candidates have been investigated only mesothelin obtained FDA approval, but its poor sensitivity limits the utility as diagnostic marker. OPN is a marker of the duration of asbestos exposure and has a potential prognostic role but lacks specificity for MPM. Other studies have shown promising results for HMGB1 (and its hyperacetylated form) and CD138 but their diagnostic role require further validation. A variety of deregulated miRNAs, as well as different new approaches based on proteomics technology, have been proposed as suitable biomarkers and are currently under investigation. Despite technical challenges, also exosomes provide a novel means of uncovering biomarkers in MPM.

As clearly emerges from this work, and from reviews published elsewhere [110–113], none of single biomarkers has ever reached adequate accuracy as to ensure its use in clinical practice. Since MPM is an extremely heterogeneous tumor, also combination of different markers together could contribute to improve diagnostic accuracy. On the other hand, clinical utility of biomarkers is dependent on how they modify subsequent clinical decisions and influence outcomes. At moment, no evidence suggests that early detection of MPM can improve survival and none of these biomarkers is recommended for screening.

More of biomarkers were studied retrospectively in small cohorts of patients, whereas analytical and pre-analytical heterogeneity affect strength of conclusions. Improvement of diagnostic accuracy, standardization of the different assays for reproducibility, choice of appropriate cut-off for each biomarker represent the main critical issues to ensure translation to daily clinical practice. Broad application of a next generation of high-performance technologies based on machine learning algorithms is expected to have a significant impact on the implementation of liquid biopsy [114]. Therefore, integration of multidisciplinary skills and increased international cooperation appears essential for improving validity of results and ensure translation to clinical practice.

## Abbreviations

AES: asbestos exposed subject
BAP1: BRCA1 associated protein
BRD: benign respiratory diseases
CD138: syndecan-1
CDKN2A: cyclin-dependent kinase inhibitor 2A
cfDNA: circulating free DNA
CTCs: circulating tumor cells
ctDNA: circulating tumor DNA
ELISA: enzyme-linked immunosorbent assay
EMT: epithelial mesenchymal transition
EpCAM: expressing epithelial cell adhesion molecule
EV: extracellular vesicles
FBLN3: fibulin-3
FOXK1: Forkhead-box K1
HMGB1: high-mobility group box 1
HS: healty subjects
miRNAs: microRNAs
MPF: megakaryocyte potentiating factor
MPM: malignant pleural mesothelioma
mRNA: messenger RNA
MS: mass spectrometry
NF2: neurofibromatosis 2
NSCLC: non-small cell lung cancer
OPD: other pleural diseases
OPE: other pleural effusions
OPN: osteopontin
OS: overall survival
PFS: progression free survival
PPV: positive predictive value
SMRP: soluble mesothelin related peptide
SPARC: secreted protein acidic and rich in cysteine
TM: thrombomodulin
VEGF: vascular endothelial growth factor
WEA: workers ex-exposed to asbestos

## Declarations

### Copyright

© The Authors 2020.
